# Impact of diabetes on gingival wound healing via oxidative stress

**DOI:** 10.1371/journal.pone.0189601

**Published:** 2017-12-21

**Authors:** Daisuke Kido, Koji Mizutani, Kohei Takeda, Risako Mikami, Takanori Matsuura, Kengo Iwasaki, Yuichi Izumi

**Affiliations:** 1 Department of Periodontology, Graduate school of Medical and Dental Sciences, Tokyo Medical and Dental University (TMDU), Tokyo, Japan; 2 Department of Nanomedicine, Graduate school of Medical and Dental Sciences, Tokyo Medical and Dental University (TMDU), Tokyo, Japan; Baker IDI Heart and Diabetes Institute, AUSTRALIA

## Abstract

The aim of this study is to investigate the mechanisms linking high glucose to gingival wound healing. Bilateral wounds were created in the palatal gingiva adjacent to maxillary molars of control rats and rats with streptozotocin-induced diabetes. After evaluating postsurgical wound closure by digital imaging, the maxillae including wounds were resected for histological examinations. mRNA expressions of angiogenesis, inflammation, and oxidative stress markers in the surgical sites were quantified by real-time polymerase chain reaction. Primary fibroblast culture from the gingiva of both rats was performed in high glucose and normal medium. *In vitro* wound healing and cell proliferation assays were performed. Oxidative stress marker mRNA expressions and reactive oxygen species production were measured. Insulin resistance was evaluated via PI3K/Akt and MAPK/Erk signaling following insulin stimulation using Western blotting. To clarify oxidative stress involvement in high glucose culture and cells of diabetic rats, cells underwent N-acetyl-L-cysteine treatment; subsequent Akt activity was measured. Wound healing in diabetic rats was significantly delayed compared with that in control rats. *Nox1*, *Nox2*, *Nox4*, *p-47*, and *tumor necrosis factor-α* mRNA levels were significantly higher at baseline in diabetic rats than in control rats. *In vitro* study showed that cell proliferation and migration significantly decreased in diabetic and high glucose culture groups compared with control groups. *Nox1*, *Nox2*, *Nox4*, and *p47* expressions and reactive oxygen species production were significantly higher in diabetic and high glucose culture groups than in control groups. Akt phosphorylation decreased in the high glucose groups compared with the control groups. Erk1/2 phosphorylation increased in the high glucose groups, with or without insulin treatment, compared with the control groups. Impaired Akt phosphorylation partially normalized after antioxidant N-acetyl-L-cysteine treatment. Thus, delayed gingival wound healing in diabetic rats occurred because of impaired fibroblast proliferation and migration. Fibroblast dysfunction may occur owing to high glucose-induced insulin resistance via oxidative stress.

## Introduction

Diabetes mellitus (DM) is a metabolic disease characterized by chronic hyperglycemia. It is a leading cause of macro- and microvascular complications [1]. Patients with diabetes have high prevalence and rate of progression of periodontal disease because of their increased susceptibility to infection [2]. Periodontal disease is characterized by local gingival inflammation due to infection with pathogenic bacteria, leading to progressive loss of alveolar bone around the involved teeth. Evidence for a bidirectional link between DM and periodontal disease has been accumulating in recent years [3–5]. Healing after dental treatment is impaired in diabetic patients. Wound healing is defective, including impairment of neutrophil activation and responses, fibroblast migration and proliferation, and angiogenesis in the diabetic condition [6, 7]. Low responses to periodontal treatment in diabetic patients have been reported [8, 9]. DM patients have elevated levels of advanced glycation end products (AGEs) in their gingival tissues [10] that may be associated with a state of enhanced oxidant stress, a potential mechanism for accelerated tissue injury. AGEs have been reported to interfere with matrix-cell interactions by altering the cross-linking of the extracellular matrix and impairing wound healing [11].

The aim of the present study was to investigate the mechanisms linking diabetes to gingival wound healing in a rodent diabetic model of insulin deficiency in comparison with nondiabetic controls. Our previous report documented that oxidative stress inhibited insulin-stimulated Akt and endothelial nitric oxide synthase (eNOS) activation in obesity-associated insulin resistance [12]. Therefore, oxidative stress in the gingiva during the wound healing process was investigated.

## Materials and methods

### Animals and induction of diabetes

**Fig 1:**
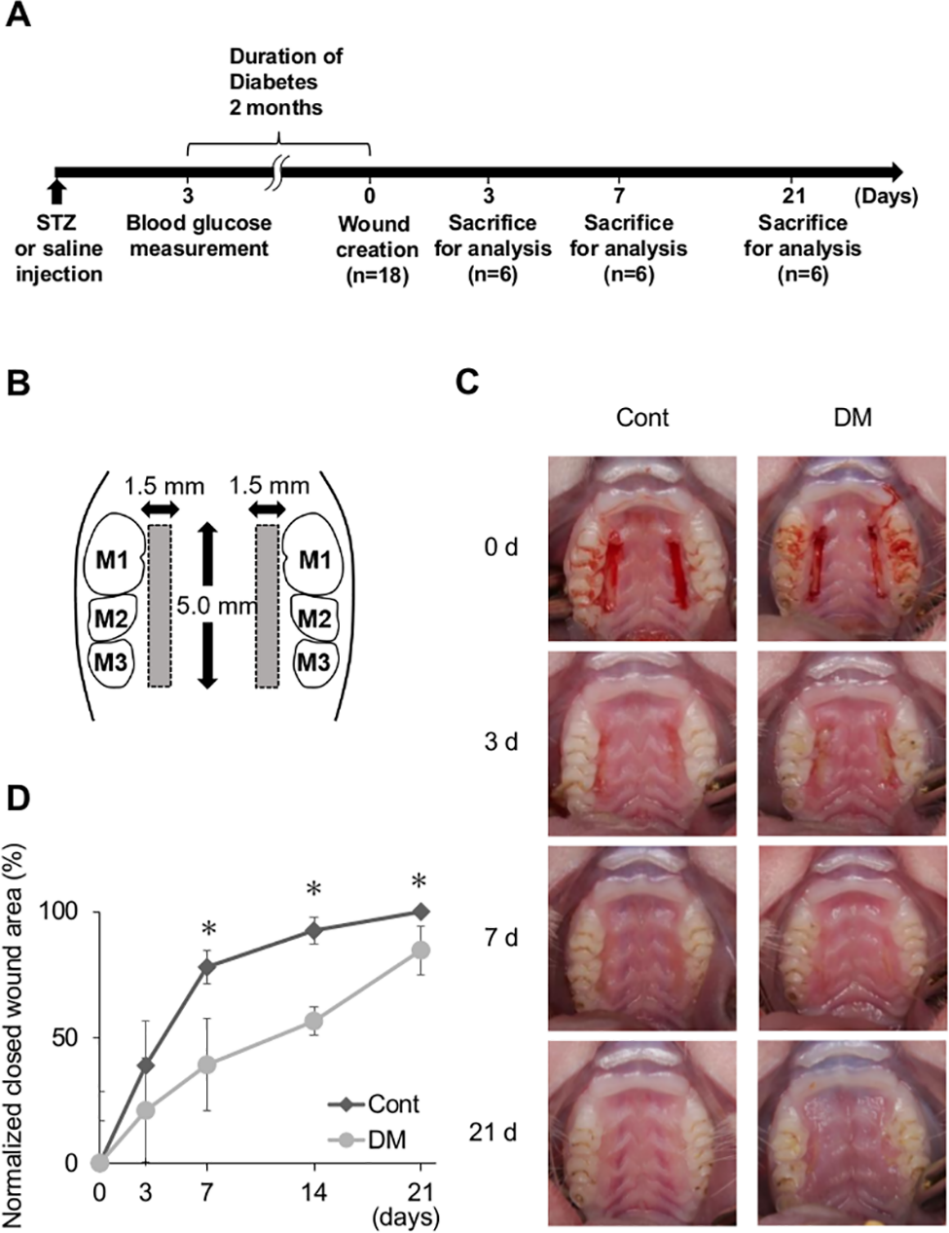
Morphometric measurements. (A) Study design. Diabetes was induced by intraperitoneal injection of streptozotocin (STZ). Seventy-two hours after injection, rats with fasting glucose levels above 350 mg/dL were used as diabetic rats. In total, 36 animals (6 per group per time point) and 72 wound sites (2 per animal) were evaluated. (B) Illustration of a palatal wound. Bilateral standardized wounds were created in the palatal gingiva adjacent to maxillary molars. M1: first molar. M2: second molar. M3: third molar. (C) Standardized photographs of wound sites at 0, 3, 7, and 21 days in control and diabetes mellitus (DM) groups. On days 7 and 21, delay of wound healing was observed in the DM group. On day 21, the wound sites were completely closed in the control group but they were still not closed in the DM group. (D) Normalized closed wound areas in control and DM groups. The area of wound closure was measured. Measurements were independently made by two blinded examiners (K.M. and K.T.), and the data were analyzed. Wound closing was delayed on days 7, 14, and 21 in the DM group. Data are presented as means ± SD (n = 6). *p < 0.05 (*t*-test).

All animal experiments were approved by the Institutional Animal Care and Use Committee of Tokyo Medical and Dental University (No. 0170207 C). The study design is illustrated in Fig 1A. Six-week-old male Slc:Wistar rats were used. The rats were fed a laboratory food (basal diet). Diabetes was induced by intraperitoneal injection of streptozotocin (60 mg/kg in saline) after 12 h of fasting [13, 14]. Seventy-two hours after injection, rats with fasting glucose levels above 350 mg/dL were used as diabetic rats [15]. Control rats (n = 18) received the same volume of saline. Body weights and plasma glucose levels were measured immediately after injection, 3 days after injection, during the surgical procedure, and at sacrifice. Blood glucose levels were measured by One Touch Ultra View® (Johnson & Johnson, NJ, USA) after 12 h of fasting. Plasma insulin levels were determined by ELISA kit following the manufacturer’s instructions (MERCODIA RAT INSULIN ELISA, MEPCODIA, Uppland, Sweden). Oxidative modified DNA in the form of 8-hydroxy-2'-deoxyguanosine (8-OHdG) can be quantified to measure the extent of DNA oxidative damage. To analyze systemic oxidative stress under a high-glucose condition, 8-OHdG in the urine was measured by ELISA kit following the manufacturer’s instructions (Japan Institute for the Control of Aging, Shizuoka, Japan).

### Creation of palatal wounds

Surgical wounds were created 2 months after injection of streptozotocin or saline. Rats were anesthetized via isoflurane inhalation. Bilateral 5.0 × 1.5-mm wounds were created in the palatal gingiva adjacent to maxillary molars as described previously with minor modifications [16], parallel to the maxillary dentition, using a No. 15 stainless steel scalpel to incise and removing the mucosal tissue, including the periosteum, with a curette (Fig 1B).

### Morphometric analysis

To evaluate wound closure, standardized intraoral digital pictures were taken immediately after the surgical procedure and on days 3, 7, 14, and 21 at a fixed magnification (×1.5) from approximately 70° to the upper occlusal plane, with the mouth opened with Hashimoto’s mouth gag (Nonaka Rikaki, Tokyo, Japan). Wound closure area was measured by digitally traced margin of un-epithelialized area using an image software (ImageJ, National Institutes of Health). Wound closure rate on each time point were evaluated based on the baseline wound area. Measurements were independently made by two blinded examiners (K.M. and K.T.), and the data were analyzed.

### Histological and histomorphometric analysis

The bilateral palatal tissues were harvested 3, 7, and 21 days after wound creation. The maxillae were fixed in 10% formaldehyde for 24 h at room temperature, decalcified with 10% ethylenediaminetetraacetic acid (EDTA) for 8 weeks at 4°C, and embedded in paraffin. To analyze re-epithelialization and the augmentation of collagen tissue, serial mesio-distal sections (thickness: 4 μm) were prepared at 1-mm intervals, stained with hematoxylin and eosin, and examined under a light microscope. For histomorphometric analysis of the wound site, the areas of epithelium and connective tissue were measured for calculation of the rate of re-epithelialization. Measurements were independently made by two blinded examiners (K.M. and K.T.), and the data were analyzed.

### Analysis of mRNA expression

The soft tissue within the wound was harvested and preserved in RNAlater® (Qiagen, Valencia, CA, USA) at baseline and on days 3, 7, and 21 after surgery. Samples were homogenized by Lysing Matrix A (MP Biomedicals, CA. USA). Total RNA was extracted with an RNeasy® Fibrous Tissue Kit (Qiagen). RNA concentration was quantified by NanoDrop Lite® (Thermo Fisher Scientific, DE, USA). Reverse transcription of total RNA was performed with a PrimeScript RT Reagent Kit (Takara, Shiga, Japan). Real-time quantitative polymerase chain reaction (PCR) was performed in the Thermal Cycler Dice® Real Time System II (Takara) with SYBR® Premix Ex Taq™ II (Takara) for *in vivo Nox1*, *Nox2*, *Nox4*, *p-47*, *eNOS*, *vascular endothelial growth factor* (*VEGF*), *tumor necrosis factor-α* (*TNF-α*), *basic fibroblast growth factor* (*FGF2*), and *collagen type I*. Gene expression levels were normalized by *glyceraldehyde 3-phosphate dehydrogenase* (*GAPDH*). Primer sequences are listed in S1 Table.

### Primary cell culture

In the surgical procedure, the resected gingiva, including the epithelium and connective tissue, was harvested from the rats. After resection, the gingival tissue was washed in phosphate-buffered saline (PBS) containing 3% antibiotics and immersed in Dulbecco’s modified Eagle’s medium (D-MEM) containing 20% dispase I for 24 h at 4°C to separate the connective tissue from the epithelial layer [17]. The connective tissue was minced into small pieces (2 mm^2^) and incubated in D-MEM with 10% fetal bovine serum (FBS) and 3% antibiotics for 2 weeks at 37°C and 5% CO_2_. Epithelial cells were not stably extracted and cultured by the methods indicated previously, and only fibroblasts were used in this study. Morphological observation of cultured cells was performed to confirm their purity following the conventional method [18]. Briefly the inspection of culture dish with phase-contrast microscope showed uniformed spindle shape at between the third and fifth passage S1 Fig, then the cells had applied for *in vitro* experiments as gingival fibroblasts [19–21].

Glucose concentrations of 5.5 mM as a control solution and 20–30 mM as a relatively high solution are used to determine the effect of high glucose on cells [22, 23]. Conversely, various glucose concentrations (e.g., 11 mM as a control solution and 50 mM as a relatively high solution) have been used depending on the cell species and purpose of the study [24–27]. In the experiments using human dermal fibroblast, cell proliferation and collagen synthesis were significantly increased in 15 mM than 5mM [28, 29]. It was reported that, in human gingival fibroblast incubated in ranging from 5 to 50 mM glucose media for 72 h, the collagen synthesis evaluated by ^3^H-proline incorporation was largest when incubated with 20 mM glucose media [30].

Prior to *in vitro* studies, we investigated the optimal glucose concentration for the primary culture of gingival fibroblasts from rats. Depending on glucose concentration, the culture media were adjusted with an appropriate volume of mannitol, for osmotic control. Cells isolated from healthy rats were cultured in 5.5 mM D+glucose + 94.5 mM D-mannitol, 15 mM D+glucose + 85 mM D-mannitol, 20 mM D+glucose + 80 mM D-mannitol, 25 mM D+glucose + 75 mM D-mannitol, 50 mM D+glucose + 50 mM D-mannitol, 75 mM D+glucose + 25 mM D-mannitol, and 100 mM D+glucose for 72 h.

After incubation, lactate dehydrogenase (LDH) release measurements and *in vitro* wound healing assay were conducted to verify cytotoxicity and cell migration. LDH release was measured in cell-free culture supernatants according to an enzymatic redox reaction, wherein tetrazolium salt is reduced to the colored product, formazan. The procedure was conducted according to the manufacturer’s protocol using a Cytotoxicity Detection Kit (LDH) (Roche, Basel, Switzerland). For the analysis, supernatants from each culture solution were added to 96-well plates and incubated with 100 μL premixed test solution (100 μL/well). The color reaction was developed for 30 min at room temperature. Light absorbance was measured at 490 nm using a 2030 ARVO MX microplate reader (Perkin Elmer, MA, USA).

An *in vitro* wound healing assay was performed to analyze the optimal glucose concentration on cultured gingival fibroblast. After cells reached 80% confluence, they were exposed to each glucose concentration for 72 h. Cells were then scored with a sterile scraper in a 1 mm wide straight line. The medium was replaced immediately after scratching to remove the debris. Every 9 h for 36 h after scratching, the plate was placed under a phase-contrast microscope; the cells and scratched area were observed and digital photographs were taken by matching the reference point. Images were analyzed by two blinded examiners (K.M. and K.T.) using the ImageJ software.

### *In vitro* wound healing assay

Based on the pilot experiments, fibroblasts from control rats (CF) and DM rats (DF) were maintained at high (75 mM glucose) or normal (15 mM glucose + 60 mM mannitol) glucose concentrations. An *in vitro* wound healing assay was performed as mentioned above. Every 6 h for 48 h after scratching, the cells and scratched area were observed as a same manner.

### Cell proliferation

The cell proliferation rate was determined by the uptake of 5-ethynil-2-deoxyuridine (EdU) into DNA using a Click-iT EdU microplate assay kit (Invitrogen, CA, USA) according to the manufacturer’s instructions. The EdU incorporated in DNA was coupled with Oregon Green-azide and subsequently incubated with horseradish peroxidase-labeled anti-Oregon Green antibody and Amplex UltraRed. The fluorescence [expressed as relative fluorescence units (RFU)] at 490 nm excitation / 585 nm emission was measured using a 2030 ARVO MX microplate reader and expressed as the cell proliferation rate [31]. CF and DF were seeded in 96-well plates at 5,000 cells per well. The cells were incubated in 15 or 75 mM glucose for at least 72 h before seeding. After seeding, cells were incubated at each glucose concentration for 8 h. Ten microliters of EdU solution was added to each well, and the plate was incubated for 16 h. Further, to count the total number of cells, EdU stained cells were incubated with 1 mg/ml Hoechst 33342 (Dojindo, Kumamoto, Japan) for 30 min at room temperature. Samples were examined under a Keyence fluorescence microscope BZ-8000 (Keyence, Osaka, Japan). Stained cells were counted in captured images and percentages of EdU stained cells were calculated. Images were analyzed by two blinded examiners (K.M. and K.T.) using the ImageJ software.

Cell proliferation analysis was also performed using a (2-(2-methoxy-4-nitrophenyl)-3-(4-nitrophenyl)-5-(2,4-disulfophenyl)-2H-tetrazolium) monosodium salt (WST-8) (Cell Counting Kit 8 [CCK-8]), Dojindo). This assay enables sensitive colorimetric assays for the determination of cell viability in cell proliferation and cytotoxicity assays. The water-soluble tetrazolium salt, WST-8, is reduced by dehydrogenase activity in cells to give a yellow formazan dye, which is soluble in the tissue culture medium. The amount of formazan dye generated by the activity of dehydrogenases in cells is directly proportional to the number of living cells. This assay is a highly established method to evaluate cell viability and is known for its high sensitivity similar to that of the MTT or the [^3^H] thymidine uptake method [32, 33]. CF and DF were seeded in 96-well plates at 5,000 cells per well. The cells were incubated in 15 or 75 mM glucose for at least 72 h before seeding. After seeding, cells were incubated at each glucose concentration for 8 h. Ten microliters of WST-8 solution was added to each well, and the plate was incubated for 2 h. Cell numbers were assessed by measuring optical absorbance on the microplate reader at 450 nm.

### *In vitro* mRNA expression analysis

Total RNA was extracted from the cells by using a RNeasy Mini Kit and Qia shredder (Qiagen). After extraction, reverse transcription and normalization were performed using the same method as in the *in vivo* study. In CF and DF, mRNA expression of the oxidative stress markers *Nox1*, *Nox2*, *Nox4*, and *p47* was quantified by real-time PCR.

### ROS production

To analyze oxidative stress under a high glucose condition, reactive oxygen species (ROS) were examined using C_27_H_19_Cl_3_O_8_ derivatives (CM-H2DCFDA; Thermo Fisher scientific) following the manufacturer’s instructions. CF and DF were incubated with 15 or 75 mM glucose solution for 72 h. Fibroblasts grown on a 96-well plates were washed and loaded with 5 μl of CM-H_2_DCFDA in D-MEM without FBS. The cells were incubated for 30 min at 37°C, and the fluorescence signal of the free radical oxidation product of CM-H_2_DCFDA, 2’,7’-dichlorofluorescein (excitation, 488 nm; emission, 525 nm), was measured using a 2030 ARVO MX microplate reader. Stained cells were captured using Keyence fluorescence microscope BZ-8000 (Keyence). And the area of CM-H2DCFDA stained cells in each well was calculated as a percentage using the captured digital images. Images were analyzed by two blinded examiners (K.M. and K.T.) using the ImageJ software.

### Analysis of insulin signaling pathway

To analyze insulin signaling-related cell migration and proliferation via the PI3K and MAPK pathways, phosphorylated Akt (p-Akt) and Erk1/2 (p-Erk1/2) following insulin stimulation (100 nM) was evaluated by Western blotting. Cells were incubated to nearly confluent with each glucose concentration in D-MEM containing 10% FBS. After 24 h of fasting, 100 nM insulin was added to each dish for 10 min. Following insulin stimulation, the medium was removed and washed by ice-cold PBS three times. Lysis buffer containing RIPA Buffer (Wako, Tokyo, Japan), protease inhibitor cocktail, and phosphatase inhibitor (Sigma, MO, USA) was added to the culture dish, and the cells were scraped with a cold scraper and collected. The pellets were sonicated and centrifuged at 13,400 rpm for 10 min at 4°C. The supernatants, 4X Laemmli sample buffer (Bio-rad, CA, USA), and 2-mercaptoethanol (Sigma) were mixed and boiled for 5 min at 95°C. Equal amounts of proteins were loaded into the Mini-Protean TGX gels (Bio-rad), and the gels were run for 40 min at a constant voltage of 100 V. After the gels were run, sample proteins were transferred to nitrocellulose membranes (Amersham) at a constant current of 200 mA for 2 h and rinsed with Ponceau solution for 1 min to check for protein transfer. Membranes were incubated for blocking at room temperature with Blocking Buffer (StartingBlock T20, Thermo Fisher Scientific) for 30 min. After blocking, the membranes were incubated overnight with a 1:1000 dilution of rabbit polyclonal primary antibody p-Akt (Cell Signaling TECHNOLOGY #4060), Akt (Cell Signaling TECHNOLOGY #9272), p-Erk (Cell Signaling TECHNOLOGY #4370), and Erk (Cell Signaling TECHNOLOGY #9102) in Blocking Buffer at 4°C. The membranes were washed with PBST (Na_2_HPO_4_ 80 mM, NaH_2_PO_4_ 50 mM, NaCl 100 mM, and 1% Tween 20) for 5 min and incubated with a 1: 5,000 dilution of donkey antirabbit IgG-HRP (Santa Cruz Biotechnology, sc-2313, TX, USA) secondary antibody in Blocking Buffer at room temperature for 1 h. After incubation, the membranes were washed intensively with PBST five times. Protein expression was normalized by comparing the signal from a monoclonal HRP-conjugated anti-*β*-actin antibody (Cell Signaling Technology #5125) at 1:1,000 dilution. For *β*-actin detection, membranes were stripped with Western BLoT Stripping Buffer® (Takara) and re-probed. The enhanced chemiluminescence reaction generated by ECL Western Blotting Substrate (Pierce) was measured using Ez-Capture MG (Atto, Tokyo, Japan) and analyzed using Image J software.

### Antioxidant treatment

The adverse effect of N-acetyl-L-cysteine (NAC) is dose dependent [34]. To measure the adequate concentration of NAC, different concentrations of NAC were determined in CF75 exposed to 0.1, 1.0, 5.0, and 10 mM NAC. We previously found that high concentrations of NAC (5 mM) prevented high glucose and insulin resistance in primary cultures of rat gingival fibroblasts S2 Fig. To analyze the connection between high glucose status and oxidative stress in the cell, an antioxidant, NAC (5 mM), was added 120 min before insulin stimulation, and the subsequent Akt activity was measured.

### Statistical analysis

Values are reported as means ± SD. Comparisons between two groups, such as control vs. DM or 15 mM vs. 75 mM, were performed using an unpaired Student *t*-test. Comparisons between multiple groups, such as between days 0, 3, and 7, were performed with one-way analysis of variance (ANOVA) followed by the Tukey-Kramer post hoc test. *P-*values less than 0.05 were considered to indicate statistically significant differences.

## Results

### Systemic characteristics of control rats and DM rats

In DM rats, body weight and plasma insulin level significantly decreased and fasting blood glucose levels significantly increased. The systemic oxidative stress levels measured by 8-OHdG in urine were significantly elevated in DM rats Table 1.

**Table 1:** Body weight, fasting blood glucose level, plasma insulin level, and urine 8-OHdG level in rats changed due to streptozotocin medication (60 mg/kg).

		Cont (n = 18)	DM (n = 18)	Significance
**Before diabetes induction**	**Body weight (g)**	**145.4 ± 9.8**	**153.7 ± 25.8**	**NS**
	**Fasting blood glucose (mg/dL)**	**126.1 ± 12.4**	**126.4 ± 12.7**	**NS**
**2 months after diabetes induction**	**Body weight (g)**	**301.4 ± 9.6**	**204.0 ± 35.0**	***p* < 0.001**
	**Fasting blood glucose (mg/dL)**	**112.6 ± 10.9**	**465.1 ± 63.7**	***p* < 0.001**
	**Plasma insulin level (μg/l)**	**80.4 ± 12.5**	**1.0 ± 0.1**	***p* < 0.001**
	**Urine 8-OHdG (ng/ml)**	**5.2 ± 2.8**	**9.1 ± 3.1**	***p* < 0.05**

### Morphometric and histological analysis

Standardized photographs showed that wound healing at 7 and 21 days was delayed in DM rats compared with control rats (Fig 1C). On day 21, the wound sites in control rats were completely closed and granulation tissue was seen in DM rats. On days 7, 14, and 21, the normalized closed wound area in DM rats was significantly less than that in control rats by 38.9 ± 18.4%, 36.0 ± 5.5%, and 15.4 ± 9.8%, respectively (Fig 1D).

**Fig 2:**
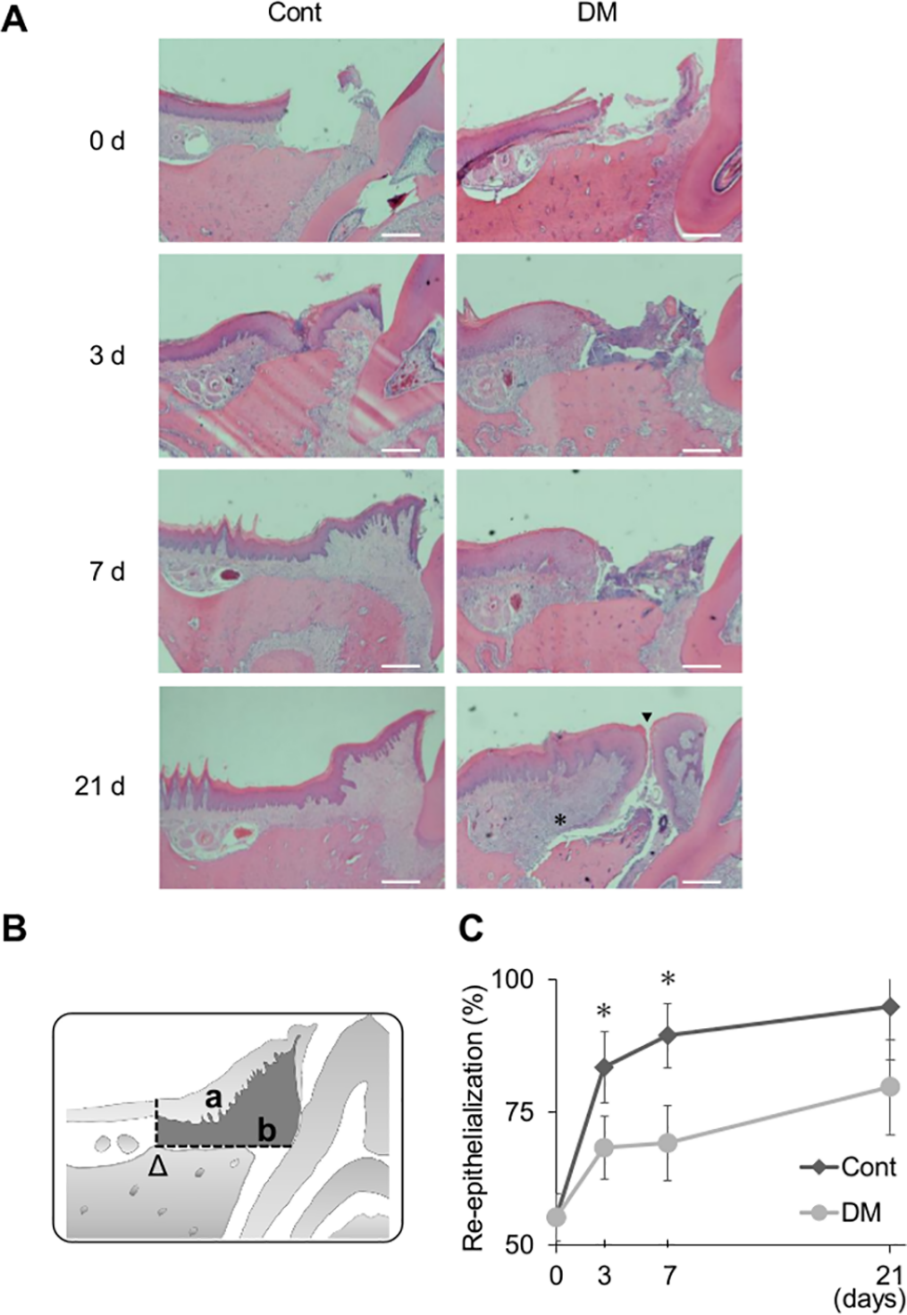
Histological and histomorphometric analysis. (A) Representative histological photographs. Note that on day 21 the wounds were completely closed and inflammatory cells were lost in the control group. While, the wounds were still not closed (arrow head) and infiltrating of inflammatory cells were not remained (asterisk) in the diabetes mellitus (DM) group. Hematoxylin–eosin staining, magnification ×40. Scale bar: 50 μm. (B) Histomorphometric analysis of wound site. The areas of epithelium (*a*) and connective tissue (*b*) were measured in the enclosed area with horizontal lines and vertical lines from the arrowhead (Δ). The re-epithelialization rate was calculated as (*a / a + b*). (C) Re-epithelialization rates in the control and DM groups. On days 3 and 7, delayed re-epithelialization was observed in the DM group. Data are presented as means ± SD (n = 6). *p < 0.05 (*t*-test).

On day 3, approximation of the epithelium was seen in the control group, whereas the exposed bone was covered with blood clot and granulation tissue in DM rats (Fig 2A). On day 7, wound closure was observed in the control group, and augmentation of the epithelium was noted in the DM group. On day 21, the wounds were completely closed and inflammatory cells were lost in the control group, whereas the wounds were still not closed in the DM group. In some DM rats, epithelial downgrowth and widespread permeation of inflammatory cells were observed. The surface of the exposed bone in DM rats was deeply stained with eosin on day 21. The re-epithelialization rate was calculated as shown in Fig 2B. Re-epithelization in the DM group was delayed by 7.6% ± 2.2% and 10.1% ± 6.5% on days 3 and 7, respectively (Fig 2C).

### *In vivo* gene expression

**Fig 3:**
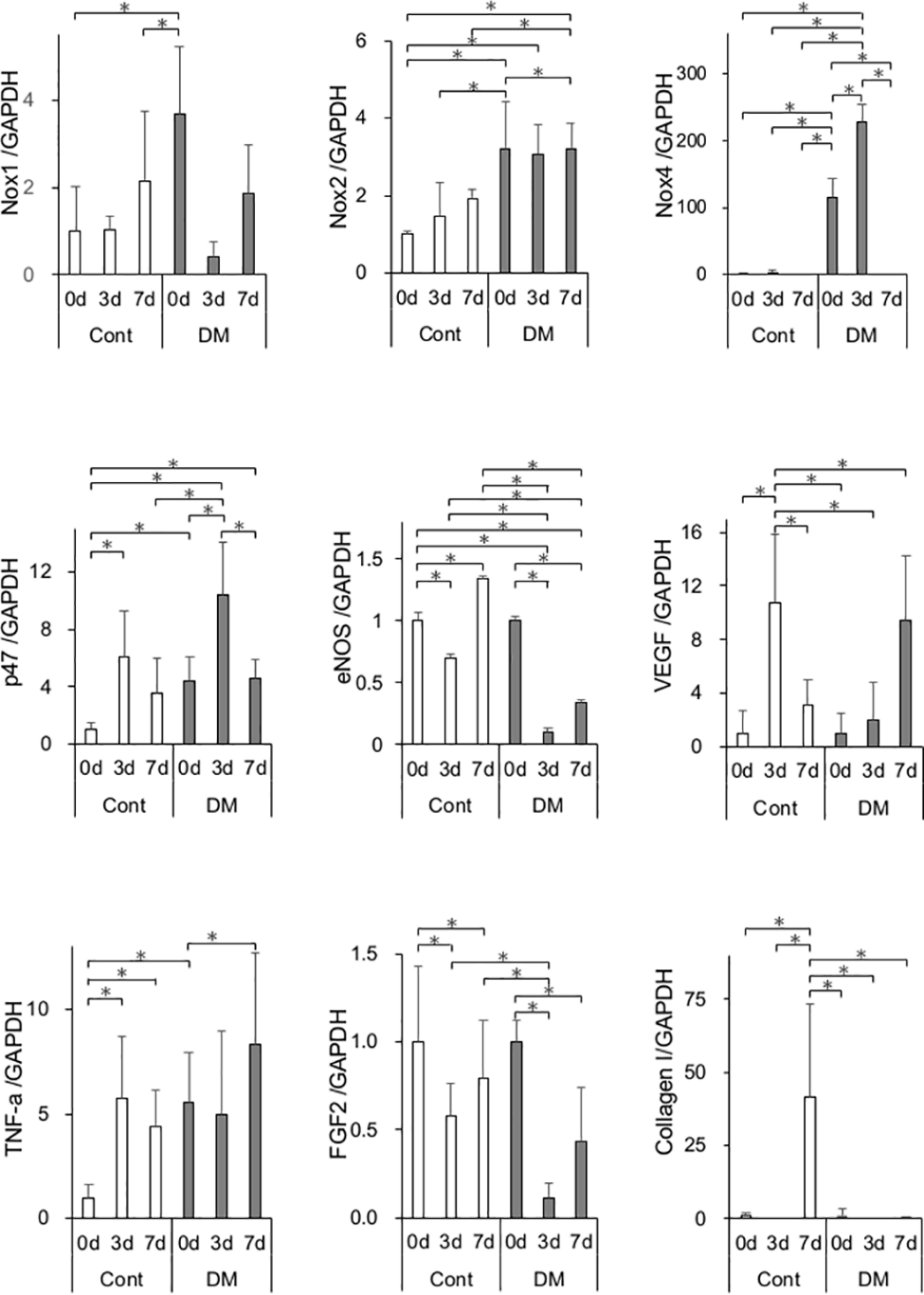
*In vivo* mRNA expression. mRNA expression of *Nox1*, *Nox2*, *Nox4*, *p47*, *eNOS*, *VEGF*, *TNF-α*, *FGF2*, and *collagen type I* genes was measured by real-time quantitative polymerase chain reaction (PCR) in the control and diabetes mellitus (DM) groups on days 0, 3, and 7. Oxidative stress markers and inflammatory cytokines were up-regulated at baseline. Expression of genes involved in angiogenesis and wound healing was significantly decreased on days 3 and 7 in the DM group. Data are presented as means ± SD (n = 6). *p < 0.05 (Tukey-Kramer test).

Expression of mRNA was analyzed (Fig 3). Expression of the oxidative stress markers *Nox1*, *Nox2*, *Nox4*, and *p47* was significantly higher in DM rats than in control rats on day 0. The same trend was observed for the inflammatory cytokine *TNF-α*. The expression of *TNF-α* was significantly higher in DM rats than in control rats on day 0. In contrast, the expression of *TNF-α* was significantly elevated in control rats on day 3. In DM rats, the expression was delayed and was significantly elevated on day 7. Both *eNOS* and *VEGF* are associated with angiogenesis. The expression of *eNOS* was significantly lower in DM rats than in control rats on days 3 and 7. The peak of *VEGF* expression was on day 3 in control rats and on day 7 in DM rats. In DM rats, the expression of *FGF2* and *collagen type I* significantly decreased on days 3 and 7, respectively.

### Primary cell culture

Fibroblasts in this study were characterized by their ability to proliferate in culture with an attached well-spread morphology S1 Fig. As assessed by phase-contrast microscope, extracted cells displayed a typical spindle shape, while epithelial cells, characterized by a cobblestone shape, were not observed. Morphometric differences were not detected in CF and DF. Microscope images were captured and analyzed by two blinded examiners (K.M. and K.T.).

**Fig 4:**
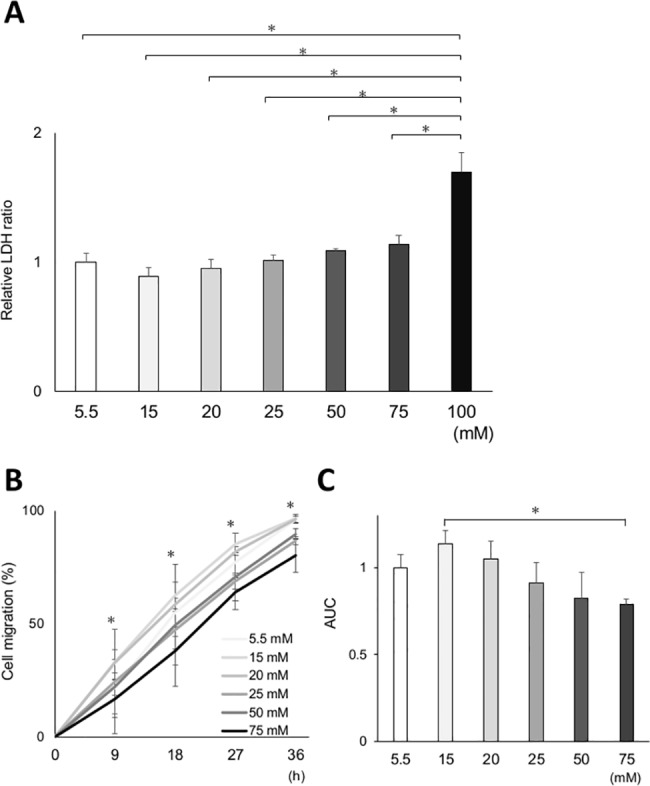
Verification of the effect of various glucose concentrations on cytotoxicity and *in vitro* wound healing assay in primary cultured rat gingival fibroblasts. Cytotoxicity and cell migration after incubation at various glucose concentrations were assessed in gingival fibroblasts from control rats. (A) Cytotoxicity was measured by lactate dehydrogenase (LDH) release in the supernatant. The results revealed a significant cytotoxicity with incubation at 100 mM glucose concentration. There was a tendency of increased cytotoxicity dose-dependent manner, although the difference was not statistically significant. (B) Effects of various glucose concentrations on *in vitro* wound healing assay. The relative cell migration area in the 75 mM group significantly decreased from 9 to 36 h compared with that in the 15 mM group. (C) The area under the curve (AUC) was calculated from the line graph. The AUC was the largest in the 15 mM group. The AUC of the 75 mM group was significantly smaller than that of the 15 mM group. Data are presented as mean ± SD. *p < 0.05 (Tukey–Kramer test). This finding was confirmed in three independent experiments.

Increased LDH release in the supernatant is an indicator of cytotoxic enzyme leakage from damaged cell membranes. Cytotoxicity at high glucose concentrations was assessed by measuring LDH release. The LDH level was lowest in 15 mM glucose media, and >20 mM glucose media slightly increased LDH release in a dose-dependent manner (Fig 4A). Exposure at 100 mM glucose media induced a significantly greater LDH release than other glucose medias (Fig 4A).

Cell migration analyzed by an *in vitro* wound healing assay more enhanced when cells were incubated in 15 mM glucose media than in 5.5 mM glucose media (Fig 4B and 4C). However, there was a dose-dependent decrease in cell migration from 20 mM to 75 mM. Cell migration was significantly impaired in 75 mM glucose media than in 15 mM glucose media. In 100 mM glucose media, cell migration could not be measured because cells were not viable after pre-incubation period. Cells incubated in 100 mM glucose media lost their attachment from the culture dish 9 h after scratch assay and floated to the medium 18 h after scratch assay.

These two results revealed that cell migration mostly increased in 15 mM glucose media and was inhibited in 75 mM glucose media with a comparable cytotoxicity at 15 mM.

### Cell migration

**Fig 5:**
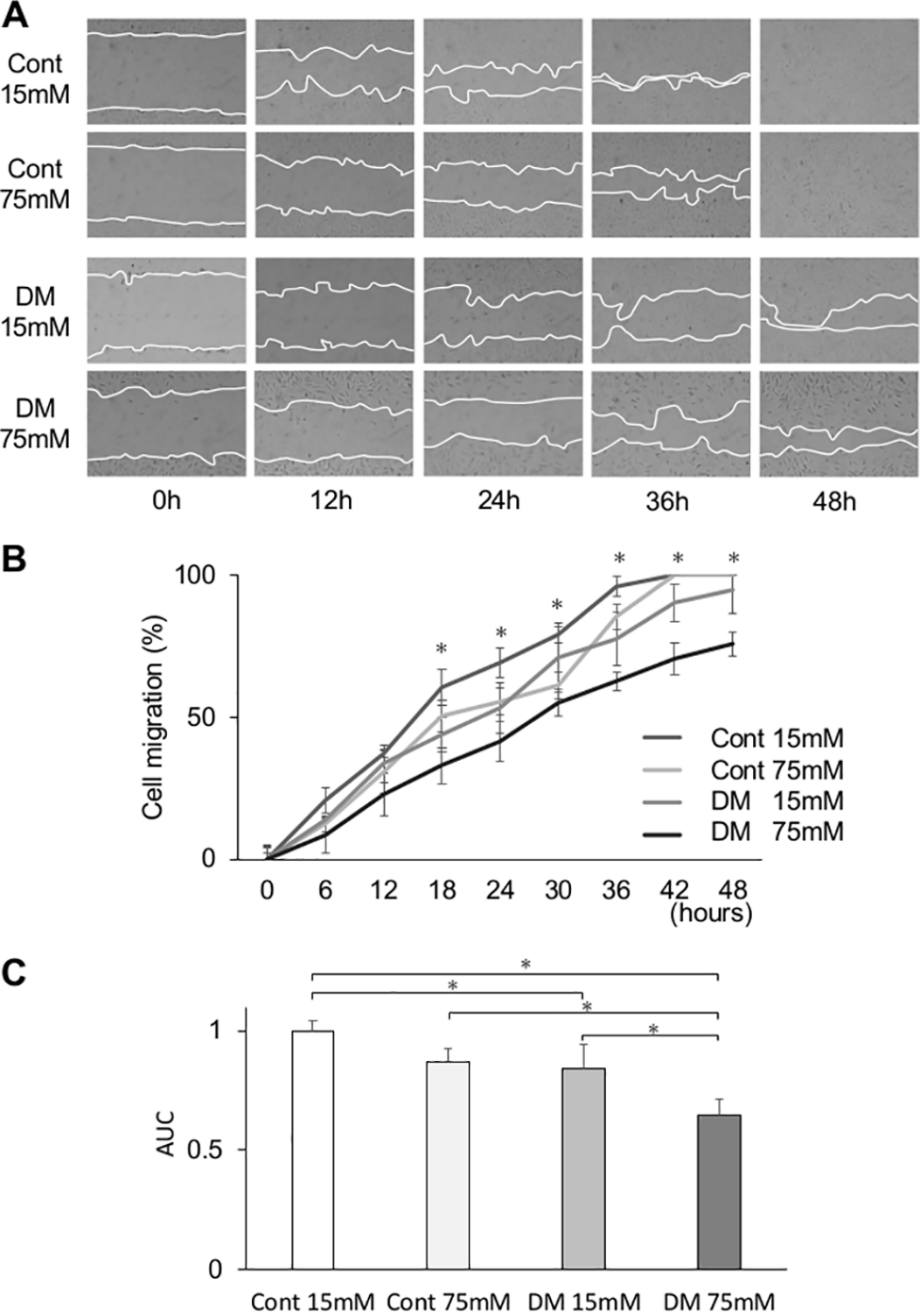
*In vitro* wound healing assay in normal and high glucose medium. (A) Representative photomicrographs of *in vitro* wound healing assay with gingival fibroblasts from control and diabetes mellitus (DM) rats. Lines show the cell migration fronts. Magnification ×40. (B) The relative cell migration area of the DM group cultured in high glucose concentration (75 mM) was significantly decreased at 18 to 48 h. Data are presented as means ± SD. *p < 0.05 (Tukey-Kramer test). This finding was confirmed in three independent experiments. (C) The area under the curve (AUC) of the relative cell migration area in the DM group was significantly low. Control cells incubated in 75 mM glucose had significantly attenuated migration compared with those incubated in 15 mM glucose at 18, 24, 30, and 36 h. DM cells incubated in 75 mM glucose also had significantly attenuated migration compared with those incubated in 15 mM glucose at 36, 42, and 48 h. DM cells incubated in control medium for 72 h had significantly decreased migration compared with control cells incubated in control medium at 18, 24, 36, and 42 h. Data are presented as means ± SD. *p < 0.05 (Tukey-Kramer test). This finding was confirmed in three independent experiments.

CF incubated in 75 mM glucose (CF75) had significantly attenuated migration compared with those incubated in 15 mM glucose (CF15) at 18, 24, 30, and 36 h (Fig 5A and 5B). DF incubated in 75 mM glucose (DF75) also had significantly attenuated migration compared with those incubated in 15 mM glucose (DF15) at 36, 42, and 48 h. However, DF15 incubated in control medium for 72 h had significantly decreased migration compared with CF15 at 18, 24, 36, and 42 h. Further, in DF75, cell migration significantly declined at 18, 24, 30, 36, 42, and 48 h compared with CF15. The area under the curve (AUC) of each cell was calculated (Fig 5C). AUC for DF75 was significantly smaller than AUC for CF15, CF75, and DF15, and AUC for DF15 was significantly smaller than AUC for CF15.

### Cell proliferation

**Fig 6:**
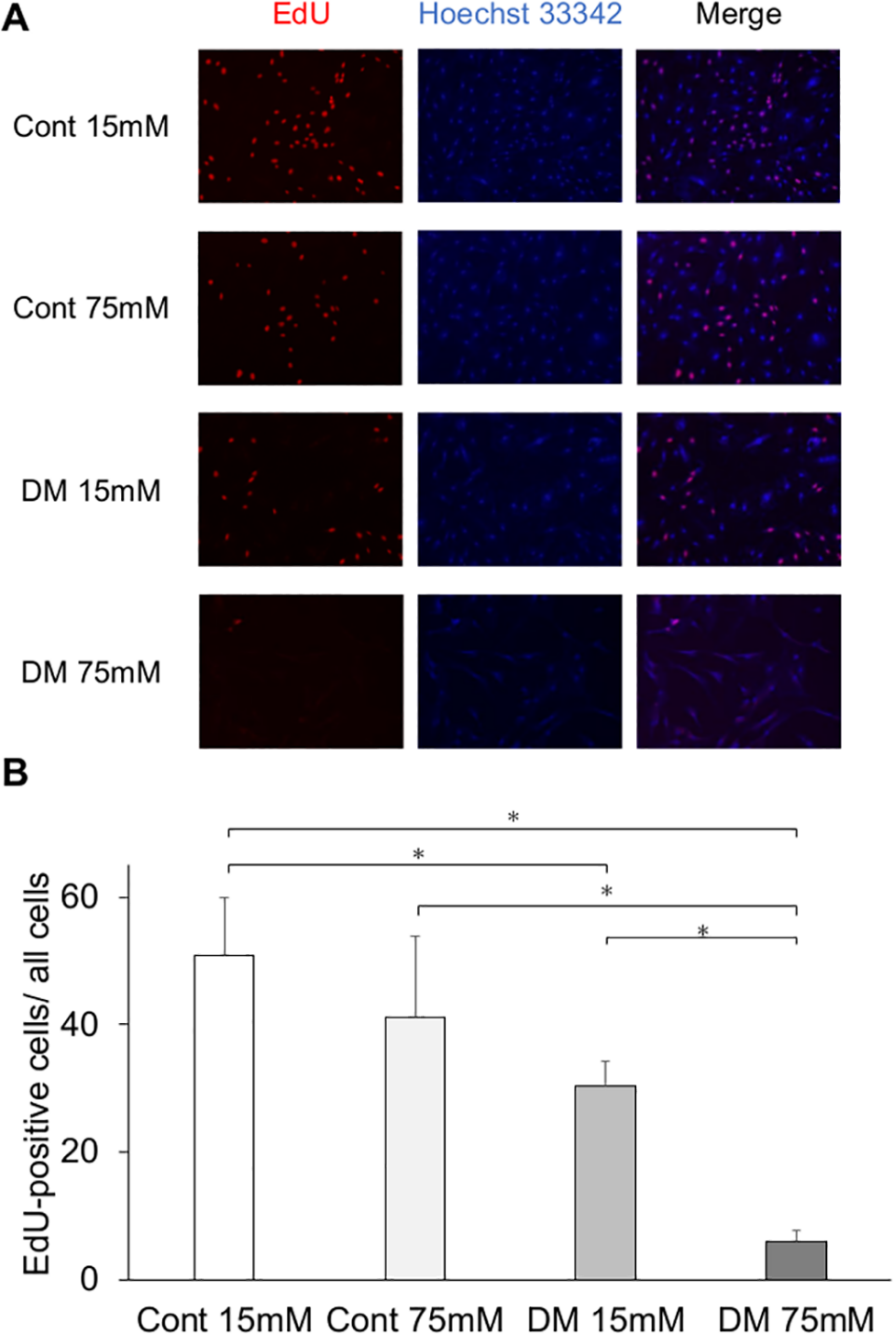
Cell proliferation analysis (EdU). Cell proliferation measured by the EdU assay was significantly less in the diabetes mellitus (DM) groups than in the control groups. (A) Representative immunofluorescence images of EdU incorporated into mitochondrial DNA, Hoechst 33342, and merged images. EdU activation was decreased in the DM groups. (B) Among the control and DM groups, cells incubated at a high glucose concentration had a significantly lower cell proliferation than those incubated at the control glucose concentration. Data are presented as mean ± SD; *p < 0.05 (Tukey–Kramer test). This finding was confirmed in four independent experiments.

**Fig 7:**
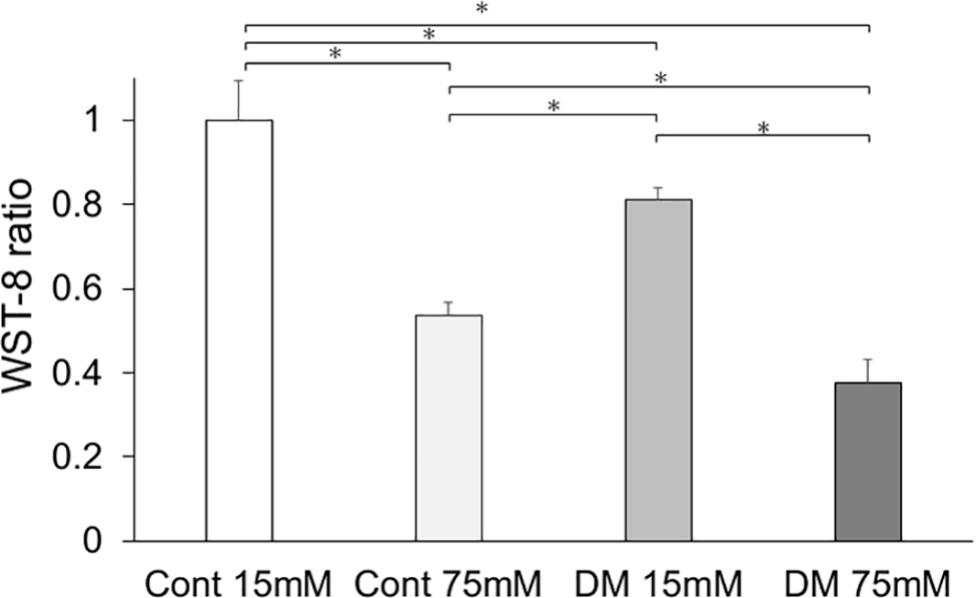
Cell proliferation analysis (WST-8). Cell proliferation measured by WST-8 assay was significantly less in the diabetes mellitus (DM) groups than in the control groups. Among the control and DM groups, cells incubated at high glucose concentration had significantly lower cell proliferation than those incubated at control glucose concentration. Data are presented as means ± SD. *p < 0.05 (Tukey-Kramer test). This finding was confirmed in four independent experiments.

We determined the effect of high glucose concentration of culture medium on the growth rate of gingival fibroblasts. In the EdU assay, the cell proliferation of DF15 and DF75 were significantly decreased compared with that of CF15 (Fig 6), whereas the proliferation of DF75 and CF75 were significantly decreased compared with that of DF15 and CF15, respectively. In the WST-8 assay, cell proliferation of CF75, DF15, and DF75 was significantly decreased compared with that of CF15, and cell proliferation of CF75 and DF75 was significantly decreased compared with that of DF15. Cell proliferation of DF75 was also significantly decreased compared with that of CF15 (Fig 7).

### *In vitro* gene expression

**Fig 8:**
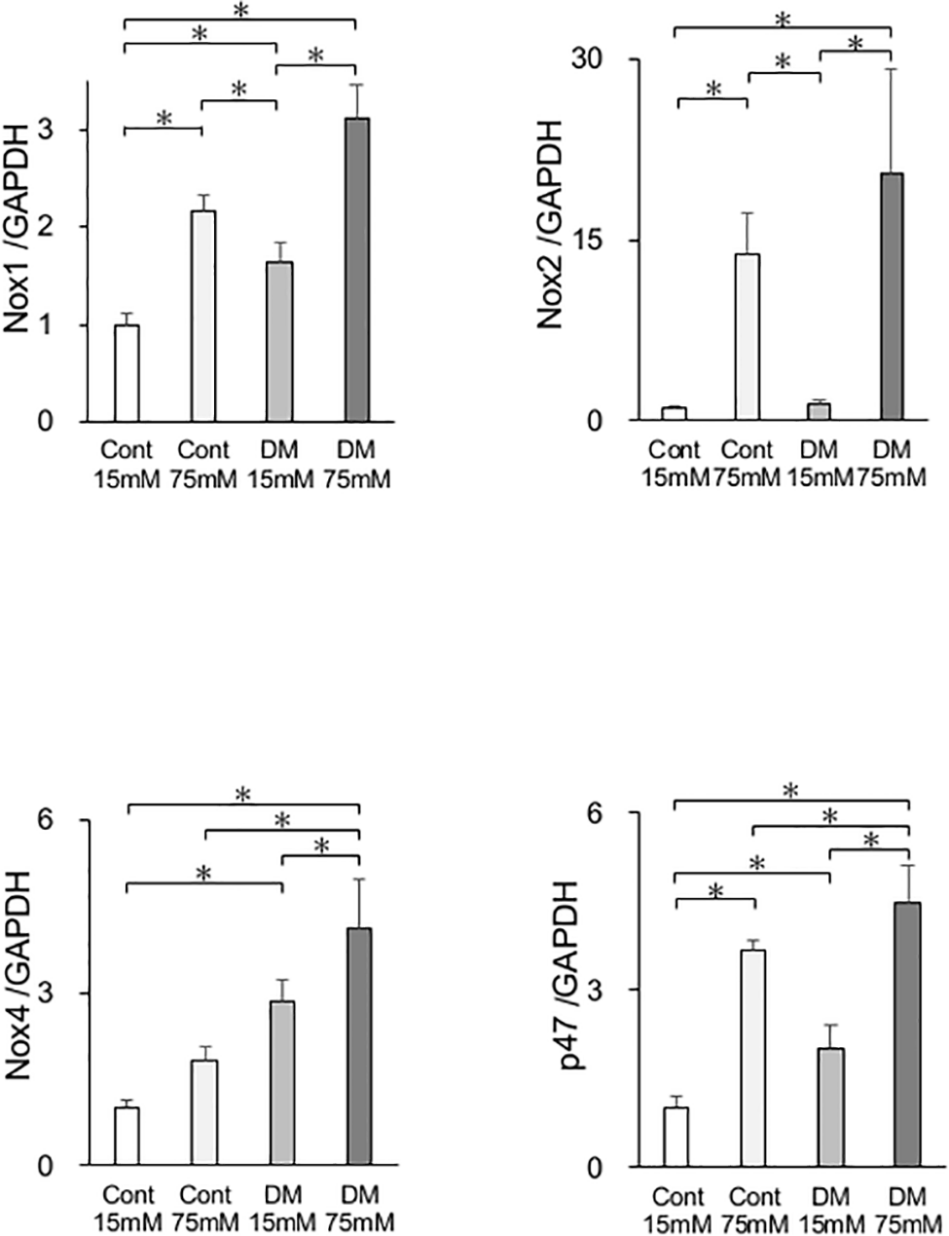
*In vitro* mRNA expression. mRNA expression of the oxidative stress markers *Nox1*, *Nox2*, *Nox4*, and *p47* in cells from control and diabetes mellitus (DM) rats was quantified by real-time quantitative polymerase chain reaction (PCR). Oxidative stress markers were significantly increased in the DM and high glucose groups. Data are presented as means ± SD. *p < 0.05 (Tukey-Kramer test). This finding was confirmed in four independent experiments.

Because expression of *Nox1*, *Nox2*, *Nox4*, and *p47* was higher in DM rats than in control rats, the expression of oxidative stress markers in CF and DF was examined (Fig 8). mRNA expression of *Nox1*, *Nox2*, *Nox4*, and *p47* in cells incubated in 75 mM glucose was significantly higher than that in cells incubated in 15 mM glucose. mRNA expression of *Nox1*, *Nox4*, and *p47* in DF incubated in 15 mM glucose was significantly higher than that in CF incubated in 15 mM.

### ROS analysis

**Fig 9:**
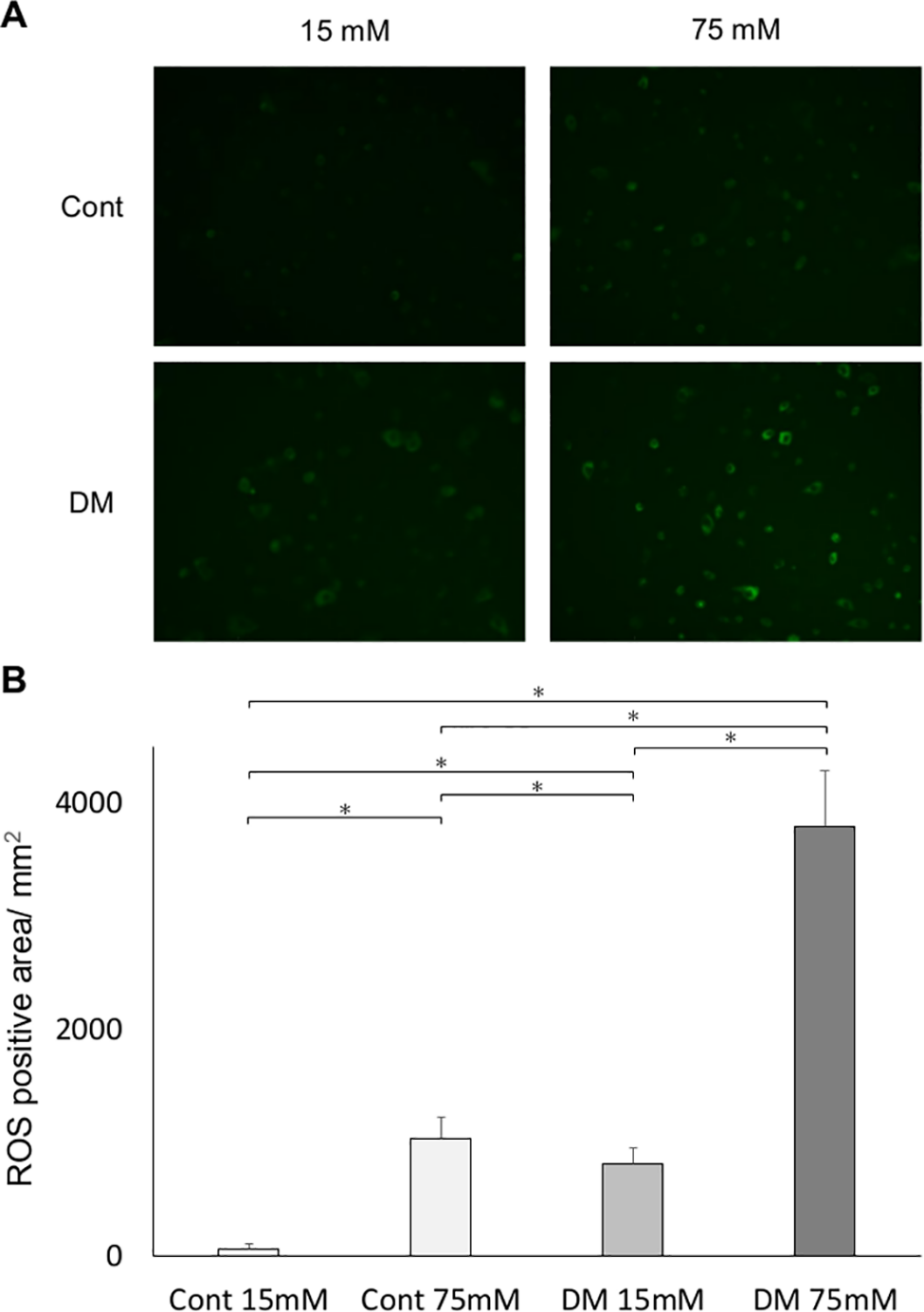
Fluorescence determination of oxidative stress. CM-H_2_DCFDA staining showed that high glucose concentration induced oxidative stress. Oxidative stress measured by CM-H_2_DCFDA assay was significantly higher in the diabetes mellitus (DM) groups than in the control groups. (A) Representative immunofluorescence images. Immunofluorescence by ROS was assessed by digital fluorescence microscopy. ROS activation was increased in the DM groups. (B) Among the control and DM groups, cells incubated at a high glucose concentration had showed a significantly higher ROS production than those incubated at the control glucose concentration. Data are presented as mean ± SD; *p < 0.05 (Tukey–Kramer test). This finding was confirmed in four independent experiments.

ROS production was evaluated by measuring the oxidation status of permeant derivatives CM-H_2_DCFDA. CM-H_2_DCFDA oxidation of DF75 and CF75 were significantly increased compared with that of DF15 and CF15, respectively. In both of CF and DF, incubation in 75 mM glucose medium caused significantly higher oxidation than that in 15mM (Fig 9).

### Insulin signaling

**Fig 10:**
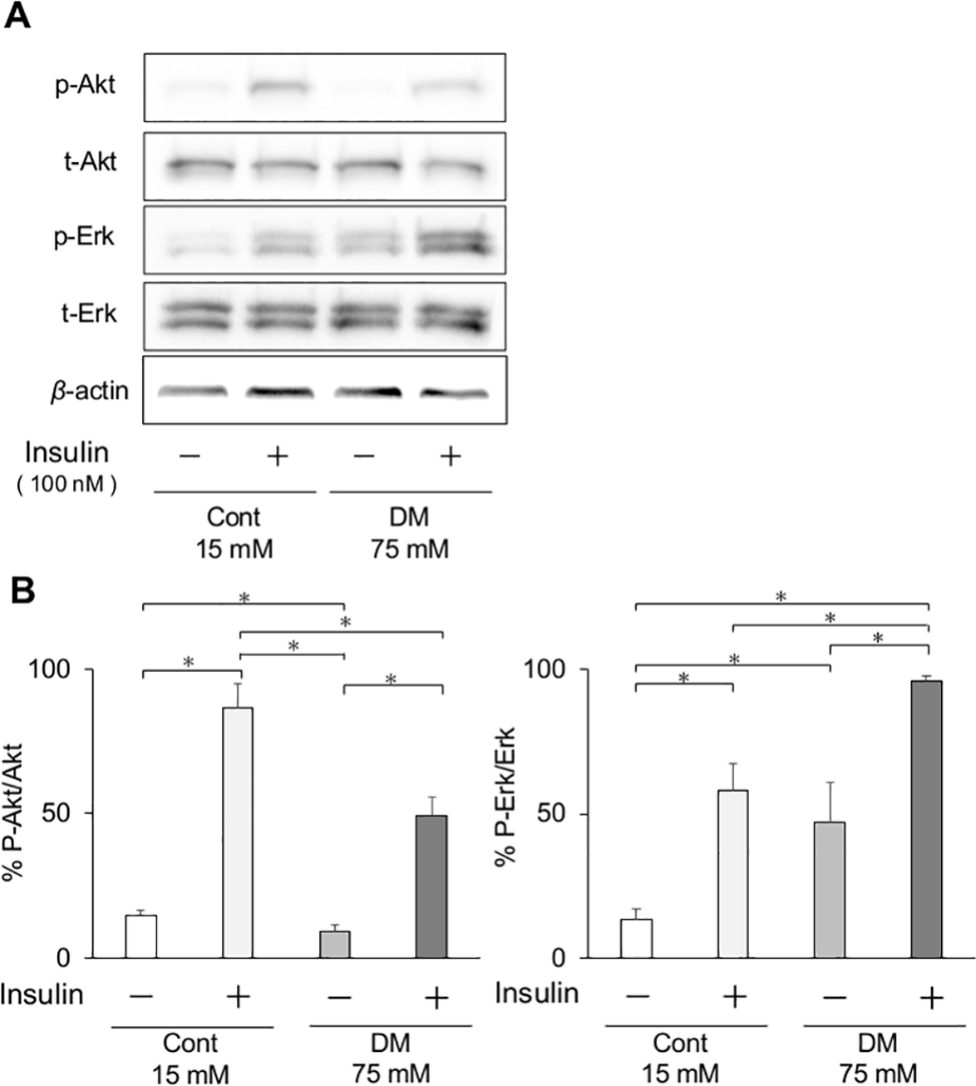
Phosphorylation of Akt and Erk1/2 in fibroblasts *in vitro*. (A) Effect of insulin on phosphorylation of Akt and Erk1/2 in gingival fibroblasts from control and diabetes mellitus (DM) rats. Representative immunoblots of lysates from insulin-treated or -untreated gingival fibroblasts were shown. (B) Phosphorylation of Akt and Erk1/2 was quantified by densitometry and expressed as percentage of phosphorylation in insulin-untreated gingival fibroblasts. Insulin-induced phosphorylation of Akt was significantly decreased in DM cells, but Erk1/2 activation was significantly increased. Data are presented as means ± SD. *p < 0.05 (Tukey-Kramer test). This finding was confirmed in three independent experiments.

Insulin-stimulated p-Akt was significantly elevated in both CF and DF (Fig 10A and 10B). Original membrane data of Fig 10 was shown in S3 Fig. In DF, insulin-stimulated p-Akt levels were significantly inhibited by 57.0 ± 7.2% compared with CF. Insulin-induced p-Erk1/2 was also elevated in both CF and DF after insulin stimulation (*p* < 0.05). In contrast to p-Akt, the basal level of p-Erk1/2 was elevated in DF without insulin stimulation. In DF, p-Erk1/2 levels were significantly increased by 165.8 ± 2.8%.

### Antioxidant treatment

**Fig 11:**
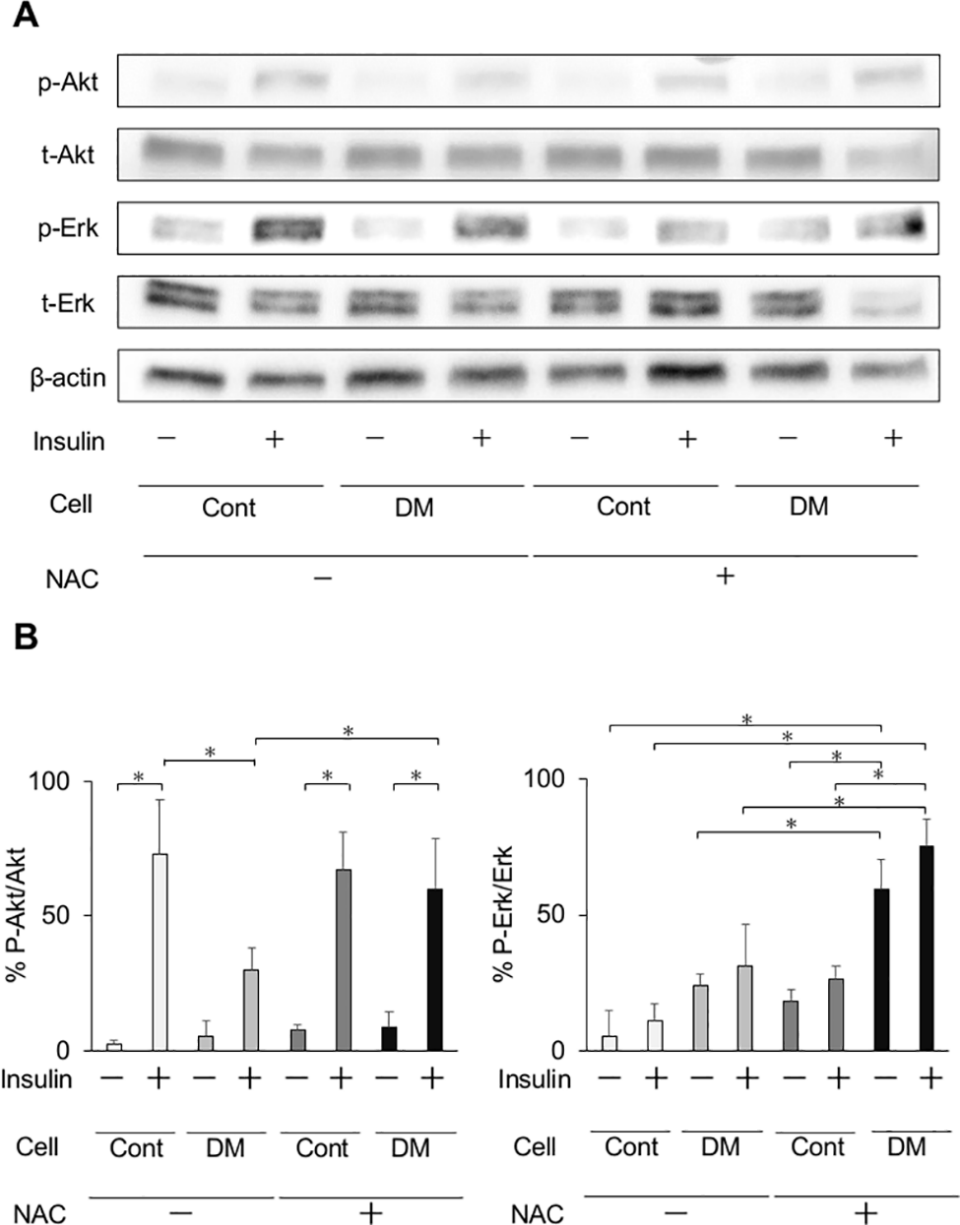
Phosphorylation of Akt and Erk1/2 in fibroblasts *in vitro* with antioxidant treatment in cells from control and DM rats. (A) Effects of treatment with the antioxidant N–acetyl–L-cysteine (NAC) on insulin signaling in gingival fibroblasts from control and diabetes mellitus (DM) rats. Representative immunoblots of lysates from insulin-treated or–untreated, and NAC-treated or -untreated gingival fibroblasts were shown. (B) Antioxidant treatment significantly improved Akt phosphorylation in DM cells. Data are presented as means ± SD. *p < 0.05 (Tukey-Kramer test). This finding was confirmed in three independent experiments.

Application of the antioxidant NAC to insulin signaling in CF15 and CF75 showed that a 5.0 mM concentration of NAC was the most effective for recovering Akt phosphorylation, which was declined by high glucose state prior to NAC treatment S2 Fig. Original membrane data of S2 Fig was shown in S5 Fig. Administration of NAC to CF and DF in the media significantly elevated Akt phosphorylation in DF (Fig 11A and 11B). Original membrane data of Fig 11 was shown in S4 Fig.

## Discussion

The present study is the first to demonstrate that wound healing in the gingiva of diabetic rats is delayed, and that poor healing is associated with impaired fibroblast function via oxidative stress. Few investigations have been reported the healing process in periodontal tissue, including the gingiva, although impaired wound healing in the diabetic state is well known. Many etiological and clinical studies have found an association between periodontitis and diabetes. It is also known that healing and tissue regeneration after nonsurgical periodontal treatment [8, 9, 35] and surgical procedures [36] are impaired in diabetic patients in dental practice. The linking mechanisms between DM and periodontitis have been mainly evaluated by the enhancement of periodontal tissue breakdown by various investigations, including damage from AGEs [37] or nitrosative stress [38] and the decreased host immune response via superoxide release [39]. Only a few *in vivo* studies have documented that AGEs are involved in the mechanisms of impaired healing following experimental intervention to periodontal tissue in the diabetic state [40]. The details of the mechanisms are still controversial. Our study confirmed that diabetes due to insulin deficiency can impair the wound healing process via insulin resistance caused by hyperglycemia.

We demonstrated that oxidative stress induced by high glucose can cause insulin resistance of gingival fibroblasts. Streptozotocin-induced DM rats are widely used for the study of diabetic complications. Streptozotocin effectively destroys pancreatic β cells and causes hyperglycemia [41], and streptozotocin-induced DM rats were simply affected by high glucose. In the present study, streptozotocin induced significant body weight loss, hyperglycemia, insulin deficiency, and increased oxidative stress. For evaluation of wound contraction, the design of the wound model was carefully considered. In the present study, a gingival wound was created with the underlying periosteum. Because of resection from the periosteum, wounds were allowed to heal only from the gingival wound margin. The pattern of wound healing and histological changes in the control rats were comparable to those in a previous study [16]. In control rats, wound healing proceeded with blood clot accumulation, inflammation, proliferation, and remodeling in approximately 7 days, but wound healing was significantly delayed in DM rats affected by high glucose. Histological study showed that extension of immature epithelium and completion of wound closure were observed on days 3 and 7 in control rats, but residual granulation was observed on days 3, 7, and 21 in DM rats.

Many previous studies have shown that systemic oxidative stress is induced by high glucose [42, 43] and may have a significant role in diabetic complications [44, 45]. In rodent models, oxidative stress can impair insulin signaling, which regulates tissue contraction and repair [46]. By a chain of mechanisms, wound healing was delayed in DM rats. Systemic oxidative stress, as measured in the urine, was significantly elevated in DM rats compared with control rats. To evaluate the effect of high glucose on fibroblast, ROS production was analyzed. ROS production was significantly increased in DF. Among CF and DF, cells incubated at a high glucose concentration had significantly increased ROS production than those incubated at control glucose concentration. Further, local levels of gingival oxidative stress and inflammation were evaluated by mRNA expression. The oxidative stress markers *Nox1*, *Nox2*, *Nox4*, and *p47* were significantly elevated in the gingiva of DM rats compared with control rats before wound creation, then *TNF-α* also showed a significant elevation in DM rats compared with control rats. Since the diabetic animal model utilized in the present study is only affected by insulin deficiency, elevated levels of both oxidative and inflammatory markers in the gingiva may be caused by hyperglycemia, not by hyperlipidemia as in the obesity-induced DM model. This finding is consistent with the results of another study stating that diabetes is characterized by a chronic low-grade inflammatory state [47]. The expression of *eNOS* was significantly reduced in DM rats on day 3, resulting in decreasing angiogenesis. Further, the expression of *VEGF*, up-regulated by *eNOS* expression, was significantly decreased on day 7, followed by delayed expression of *eNOS*. In the analysis of angiogenesis expression, postsurgical expression of *eNOS* and *VEGF* were significantly lower or delayed in the DM groups compared with controls. Both *eNOS* and *VEGF* are up-regulated via the insulin receptor/PI3K/Akt pathway. This finding is consistent with insulin resistance showing an impairment of Akt activation following *in vitro* experiments. The expression of *FGF2* on day 3 and of *collagen type I* on day 7 was significantly lower in DM groups than in control groups. These data reflect the abnormal cell proliferation and delayed wound healing in DM rats. Katagiri et al. reported that dermal wound healing was delayed in the high glucose state through the impaired IRS-1/Akt pathway [48]. They also observed lower angiogenesis potentially with *VEGF* expression in streptozotocin-induced diabetic mice than in control groups.

High glucose concentrations are known to affect cell function based on cell types, tissues, and organs. Cell culturing conditions occasionally differ from *in vivo* physiological conditions. Blood glucose concentrations of healthy and STZ-induced diabetic rats in this study were approximately 7 mM and 26 mM, respectively. Conversely, in this *in vitro* study, the optimal glucose concentration for cell migration was 15 mM for cultured gingival fibroblasts. Even in 75 mM glucose media, a comparable cell migration was observed, and cytotoxicity was not statistically different from that in 15 mM glucose medium. To clarify the effect of high glucose concentrations on the cultured gingival fibroblasts, we selected the highest possible, biologically applicable glucose concentration without inducing cytotoxicity. In this study, 15 mM glucose was used as a control, and 75 mM glucose was used for the test group. Willershausen et al. reported that collagen synthesis of human cultured gingival fibroblast was the most promoted in 20 mM glucose, and significantly decreased in 25 mM and 50 mM [30]. Within the limitation such as animal species, it was consistently considered that the optimal glucose concentration for *in vitro* gingival fibroblast experiments was higher than that of *in vivo*.

To replicate the result of an *in vivo* study, cell proliferation and migration were analyzed by an *in vitro* wound healing assay, EdU assay, and a WST-8 assay. Consistent with the previous study, fibroblast proliferation [49, 50] and migration [6, 51] were significantly decreased in DF compared with CF. We performed cell cycle analysis using the EdU assay and enzyme activity analysis for indirect viable cell counting using the WST-8 assay and found that cell proliferation was decreased in cells from DM rats and in those incubated at a high glucose concentration. Additionally, the rates of cell migration were significantly decreased by incubation in 75 mM glucose in both CF groups and DF groups. Previous studies showed that fibroblast proliferation and migration are prerequisites for wound healing [52–54]. Thus, it is assumed that wound healing was delayed in DF due to the impaired cell proliferation and migration. In the present study, glucose concentrations of 15 mM and 75 mM were used as the control and high glucose states, because 10 to 20 mM seems to be optimal for fibroblast culture, and more than 50 mM impairs proliferation [30]. Consequently, these concentrations indicated the characteristics for the glucose concentration in rat fibroblasts. Interestingly, when DF were incubated in 15 mM glucose for 72 h, cell proliferation and migration capabilities did not completely recover. A previous study showed that epigenetic manifestations [55] and protein kinase C activation [56, 57] were induced by an extended high glucose state. In the same way, some irreversible changes were induced by 8 weeks of hyperglycemia. Furthermore, the oxidative stress markers *Nox1*, *Nox2*, *Nox4*, and *p47* in mRNA level were increased in DF, and consequently, it was suggested that oxidative stress is elevated in the gingival tissue by hyperglycemia. Analysis of the insulin signal involved in tissue repair showed the attenuation of cell responses to insulin stimulation in DM rats. Nevertheless, in general, insulin resistance is induced by excess weight [58], physical inactivity [59], and a pathological condition in which cells do not respond properly to insulin.

To date, no experimental study has analyzed the underlying mechanisms of impaired wound healing of periodontal tissue in the diabetic state. This study demonstrates that diabetes caused by insulin deficiency can induce abnormality in the proliferation of gingival fibroblasts, which is related to the resistance of Akt phosphorylation induced by insulin. Interestingly, NAC treatment could partially recover the impaired Akt activation following insulin stimulation. It might be assumed that the increased oxidative stress in hyperglycemia is potentially involved with persistent Akt-selective insulin resistance. Insulin resistance observed in diabetes has been associated with increased risks of major diabetic complications, such as cardiovascular disease, renal dysfunction, and chronic kidney disease [60]. We assume that insulin resistance may play a key role in the worsening of periodontitis in patients with metabolic syndrome or diabetes. Further investigations of insulin resistance in periodontal tissue, including endothelial cells as well as diabetic microvascular complications, are needed to clarify the relationship between diabetes and periodontal disease and to develop a therapeutic approach for diabetic patients with periodontitis.

In conclusion, delayed gingival wound healing in diabetic rats was caused by impaired proliferation and migration of fibroblasts. Dysfunction of the fibroblasts may be caused by high glucose-induced insulin resistance via oxidative stress.

## Supporting information

S1 Table Primer sequence for PCR. (TIFF)

S1 Fig Morphological observation of the isolated gingival fibroblasts. Cells were cultured from resected palatal gingival tissue. Cells were obtained from resected gingiva of control and DM rats, and incubated in 15 mM and 75 mM glucose concentrations respectively. Cells shown at passage 3–5. Original magnification: × 40. Scale bar: 50 μm.
(TIFF)

S2 Fig Verification of effect of antioxidant concentration on phosphorylation of Akt and Erk1/2 of fibroblasts *in vitro* in cells from control and diabetes mellitus (DM) rats. Effects of treatment with the antioxidant N–acetyl–L-cysteine (NAC) on insulin signaling in gingival fibroblasts from control rats. Antioxidant treatment significantly improved Akt phosphorylation at concentrations of 5 mM and 10 mM. Data are presented as means ± SD. *p < 0.05 (Tukey-Kramer test).
(TIFF)

S3 Fig Original membrane in Fig 10. The membrane was separated at 50 kD. The membrane fragment including 60 kD was applied total and phosphorylated Akt. The other fragment including at 42, 44, and 45 kD was applied total and phosphorylated Erk1/2 and β-actin.
Membranes in two independent experiments were developed at the same time.
A. Original membrane in Fig 10, B-a. Membrane in Fig 10 as t-Akt, B-b. Membrane in Fig 10 as p-Erk1/2, C-a. Membrane in Fig 10 as p-Akt, C-b. Membrane in Fig 10 as t-Erk1/2, D-a. Membrane in Fig 10 as β-actin.
(TIFF)

S4 Fig Original membrane in Fig 11. The membrane was separated at 50 kD. The membrane fragment including 60 kD was applied total and phosphorylated Akt. The other fragment including at 42, 44, and 45 kD was applied total and phosphorylated Erk1/2 and β-actin.
A. Original membrane in Fig 11, B. Membrane in Fig 11 as p-Akt and p-Erk1/2, C. Membrane in Fig 11 as t-Akt and t-Erk1/2, D. Membrane in Fig 11 as β-actin.
(TIFF)

S5 Fig Original membrane in S2 Fig. The membrane was separated at 50 kD. The membrane fragment including 60 kD was applied total and phosphorylated Akt. The other fragment including at 42 kD and 44 kD was applied total and phosphorylated Erk1/2.
Membranes in two independent experiments were developed at the same time.
A, B. Original membrane in S2 Fig, C-a. Membrane in S2 Fig as p-Akt, C-b. Membrane in S2 Fig as p-Erk1/2, D-a. Membrane in S2 Fig as t-Akt, D-b. Membrane in S2 Fig as t-Erk1/2.
(TIFF)
