# Supplementary figures and images for: Impact of diabetes on gingival wound healing via oxidative stress
Source: PLoS One. 2017 Dec 21;12(12):e0189601. doi: 10.1371/journal.pone.0189601 (PMC5739411; doi:10.1371/journal.pone.0189601)

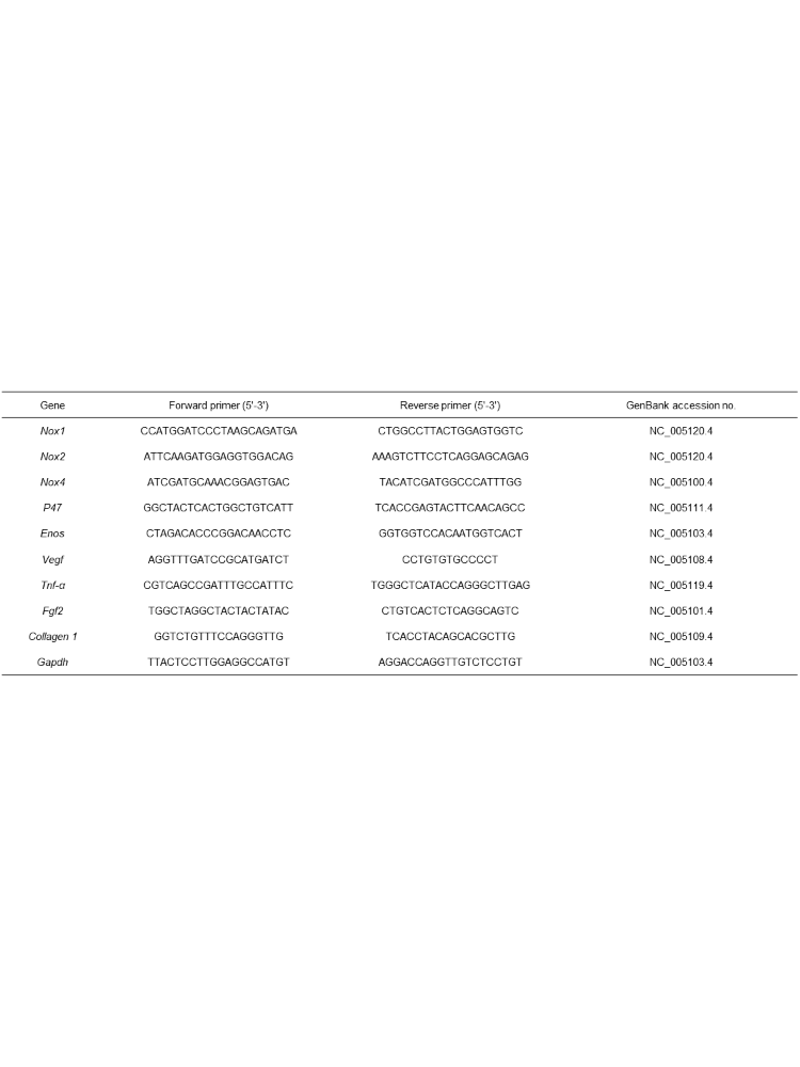

Supplement: S1 Table — (TIFF) [file pone.0189601.s001.tiff]

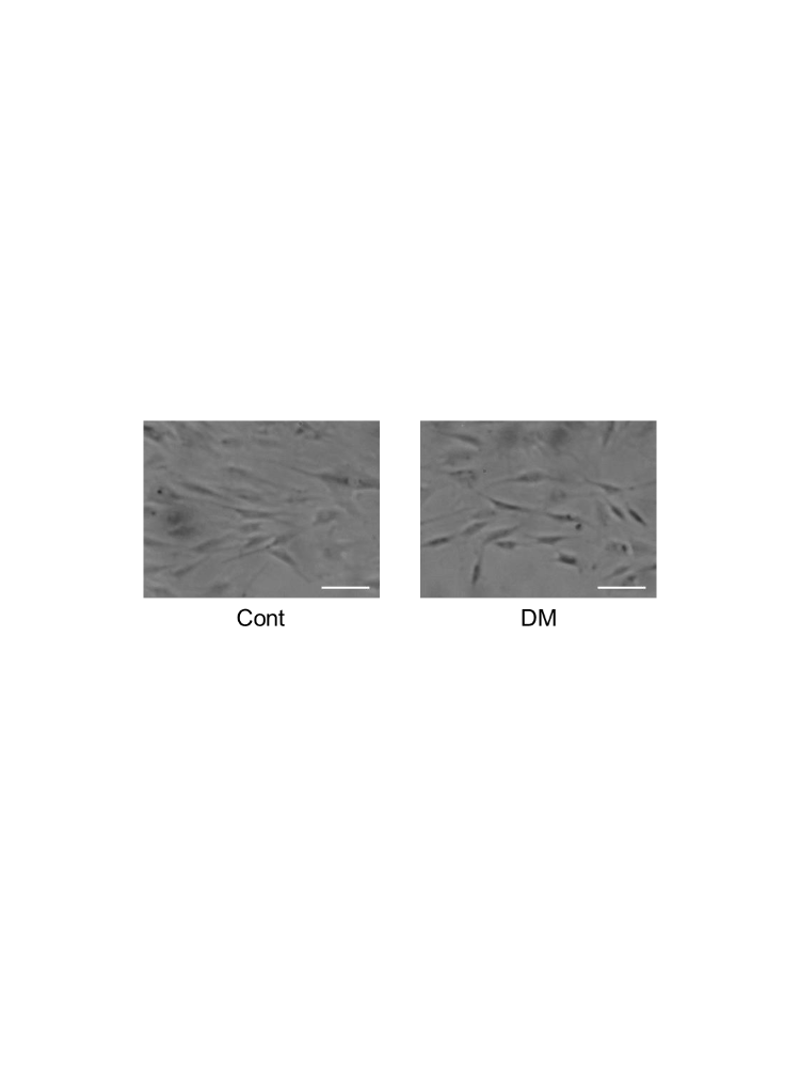

Supplement: S1 Fig — Cells were cultured from resected palatal gingival tissue. Cells were obtained from resected gingiva of control and DM rats, and incubated in 15 mM and 75 mM glucose concentrations respectively. Cells shown at passage 3–5. Original magnification: × 40. Scale bar: 50 μm. (TIFF) [file pone.0189601.s002.tiff]

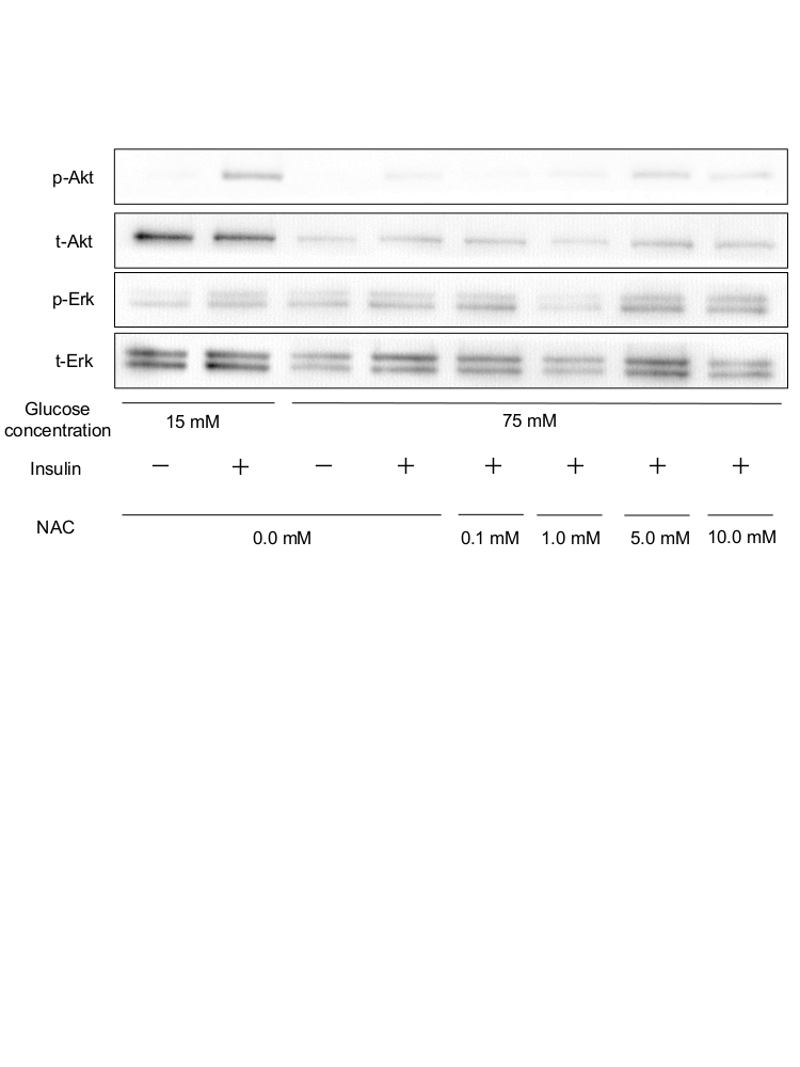

Supplement: S2 Fig — Effects of treatment with the antioxidant N–acetyl–L-cysteine (NAC) on insulin signaling in gingival fibroblasts from control rats. Antioxidant treatment significantly improved Akt phosphorylation at concentrations of 5 mM and 10 mM. Data are presented as means ± SD. *p < 0.05 (Tukey-Kramer test). (TIFF) [file pone.0189601.s003.tiff]

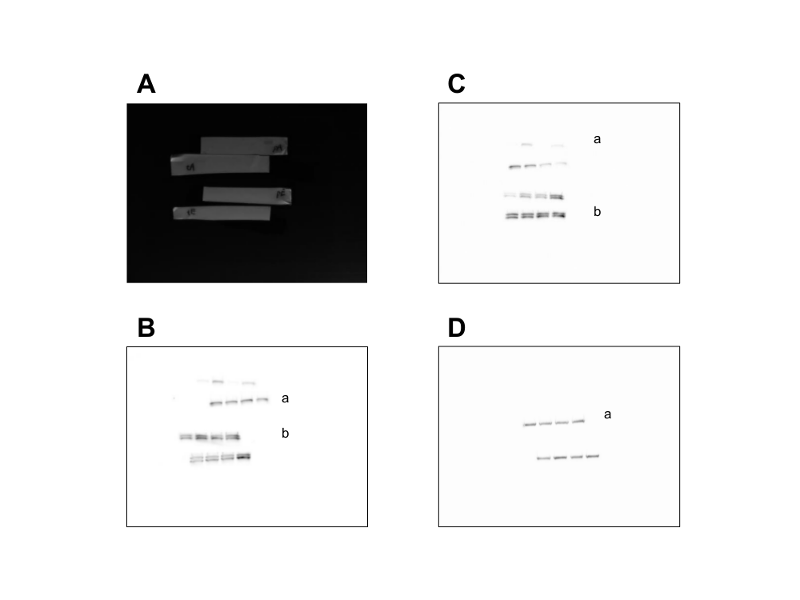

Supplement: S3 Fig — The membrane was separated at 50 kD. The membrane fragment including 60 kD was applied total and phosphorylated Akt. The other fragment including at 42, 44, and 45 kD was applied total and phosphorylated Erk1/2 and β-actin. Membranes in two independent experiments were developed at the same time. A. Original membrane in Fig 10, B-a. Membrane in Fig 10 as t-Akt, B-b. Membrane in Fig 10 as p-Erk1/2, C-a. Membrane in Fig 10 as p-Akt, C-b. Membrane in Fig 10 as t-Erk1/2, D-a. Membrane in Fig 10 as β-actin. (TIFF) [file pone.0189601.s004.tiff]

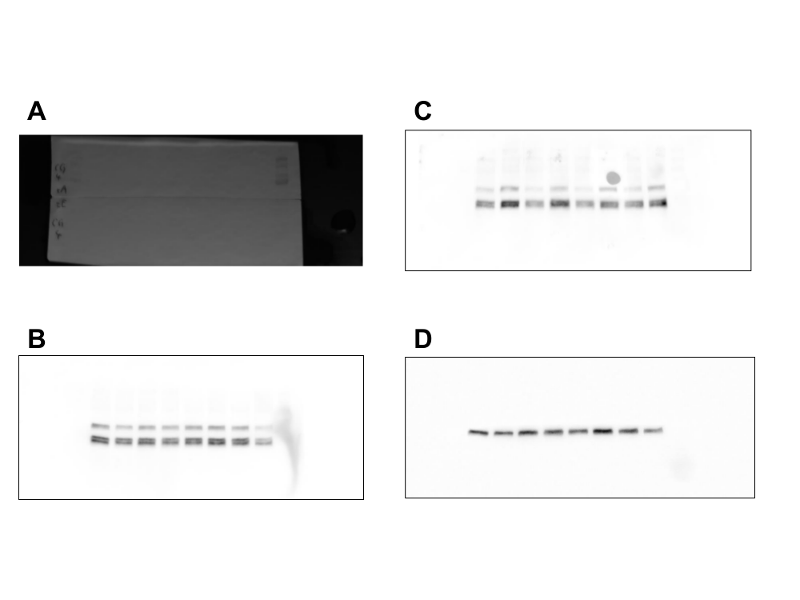

Supplement: S4 Fig — The membrane was separated at 50 kD. The membrane fragment including 60 kD was applied total and phosphorylated Akt. The other fragment including at 42, 44, and 45 kD was applied total and phosphorylated Erk1/2 and β-actin. A. Original membrane in Fig 11, B. Membrane in Fig 11 as p-Akt and p-Erk1/2, C. Membrane in Fig 11 as t-Akt and t-Erk1/2, D. Membrane in Fig 11 as β-actin. (TIFF) [file pone.0189601.s005.tiff]

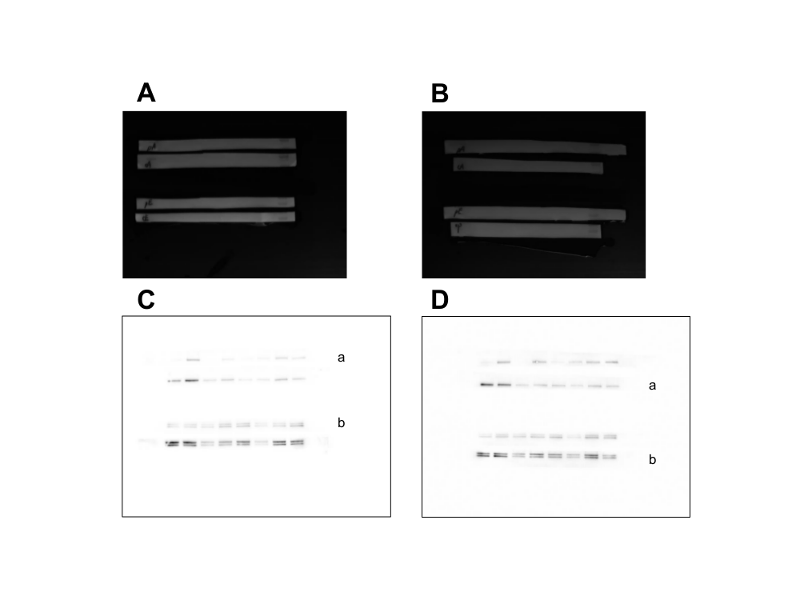

Supplement: S5 Fig — The membrane was separated at 50 kD. The membrane fragment including 60 kD was applied total and phosphorylated Akt. The other fragment including at 42 kD and 44 kD was applied total and phosphorylated Erk1/2. Membranes in two independent experiments were developed at the same time. A, B. Original membrane in S2 Fig, C-a. Membrane in S2 Fig as p-Akt, C-b. Membrane in S2 Fig as p-Erk1/2, D-a. Membrane in S2 Fig as t-Akt, D-b. Membrane in S2 Fig as t-Erk1/2. (TIFF) [file pone.0189601.s006.tiff]
